# Adapting mark-recapture methods to estimating accepted species-level diversity: a case study with terrestrial Gastropoda

**DOI:** 10.7717/peerj.13139

**Published:** 2022-06-21

**Authors:** Gary Rosenberg, Kurt Auffenberg, Ruud Bank, Rüdiger Bieler, Philippe Bouchet, David Herbert, Frank Köhler, Thomas A. Neubauer, Eike Neubert, Barna Páll-Gergely, Ira Richling, Simon Schneider

**Affiliations:** 1 Malacology Department, Academy of Natural Sciences, Philadelphia, Pennsylvania, United States; 2 Biodiversity, Earth and Environmental Science, Drexel University, Philadelphia, Pennsylvania, United States; 3 Florida Museum of Natural History, University of Florida, Gainesville, Florida, United States; 4 Medical Center Groningen, University of Groningen, Groningen, Netherlands; 5 Negaunee Integrative Research Center, Field Museum of Natural History, Chicago, Illinois, United States; 6 Institut Systématique Evolution Biodiversité, Muséum National d’Histoire Naturelle, Paris, France; 7 Department of Natural Sciences, National Museum of Wales, Cardiff, United Kingdom; 8 Malacology Department, Australian Museum Research Institute, Australian Museum, Sydney, New South Wales, Australia; 9 Department of Animal Ecology and Systematics, Justus Liebig University, Giessen, Germany; 10 Naturalis Biodiversity Center, Leiden, The Netherlands; 11 Natural History Museum Bern, Bern, Switzerland; 12 Institute of Ecology and Evolution, University of Bern, Bern, Switzerland; 13 Plant Protection Institute, Centre for Agricultural Research, Budapest, Hungary; 14 Stuttgart State Museum of Natural History, Stuttgart, Germany; 15 CASP, Cambridge, United Kingdom; 16 SNSB - Bavarian State Collection for Paleontology and Geology, Munich, Germany

**Keywords:** Species richness, Diversity, Mark-recapture, Terrestrial gastropods, Taxonomic databases, Sources of uncertainty, Global species databases, Mollusca, Biodiversity informatics

## Abstract

We introduce a new method of estimating accepted species diversity by adapting mark-recapture methods to comparisons of taxonomic databases. A taxonomic database should become more complete over time, so the error bar on an estimate of its completeness and the known diversity of the taxon it treats will decrease. Independent databases can be correlated, so we use the time course of estimates comparing them to understand the effect of correlation. If a later estimate is significantly larger than an earlier one, the databases are positively correlated, if it is significantly smaller, they are negatively correlated, and if the estimate remains roughly constant, then the correlations have averaged out. We tested this method by estimating how complete MolluscaBase is for accepted names of terrestrial gastropods. Using random samples of names from an independent database, we determined whether each name led to a name accepted in MolluscaBase. A sample tested in August 2020 found that 16.7% of tested names were missing; one in July 2021 found 5.3% missing. MolluscaBase grew by almost 3,000 accepted species during this period, reaching 27,050 species. The estimates ranged from 28,409 ± 365 in 2021 to 29,063 ± 771 in 2020. All estimates had overlapping 95% confidence intervals, indicating that correlations between the databases did not cause significant problems. Uncertainty beyond sampling error added 475 ± 430 species, so our estimate for accepted terrestrial gastropods species at the end of 2021 is 28,895 ± 630 species. This estimate is more than 4,000 species higher than previous ones. The estimate does not account for ongoing flux of species into and out of synonymy, new discoveries, or changing taxonomic methods and concepts. The species naming curve for terrestrial gastropods is still far from reaching an asymptote, and combined with the additional uncertainties, this means that predicting how many more species might ultimately be recognized is presently not feasible. Our methods can be applied to estimate the total number of names of Recent mollusks (as opposed to names currently accepted), the known diversity of fossil mollusks, and known diversity in other phyla.

## Introduction

Understanding the magnitude of biological diversity has been a goal of systematic biology, evolution and ecology since the founding of those disciplines. Yet we do not know even within an order of magnitude how many species currently exist on Earth, which makes it difficult to state how many species are at risk of going extinct when current extinction rates are estimated to be 1,000 times the background rate (Pimm et al., 2014; Andermann et al., 2020; Neubauer et al., 2021; Cowie, Bouchet & Fontaine, 2022). Estimates of the number of species of eukaryotes range from three to hundreds of millions, with most implying that the majority of species have yet to be discovered (Mora et al., 2011; Larsen et al., 2017). Estimates that include bacterial species range into the billions and as high as one trillion (Locey & Lennon, 2016; Larsen et al., 2017). Estimates of diversity that take into account abundance and disparity are even more difficult, although mathematical sophistication is rapidly increasing in treating assemblages of taxa in defined areas (Leinster & Cobbold, 2012; Chao et al., 2014).

We tackle here a simpler question: how to estimate how many species have already been named and are currently accepted. Even in this restricted domain, much uncertainty remains. The Catalogue of Life (2020) aims to attain a complete list of accepted species, by adding together global species databases (GSDs) (Bisby, 2000; Garnett et al., 2020). Costello (2020) stated that it is “about 85% complete”, as of 2019 listing 1.3 million species of 1.5 million known (based on Costello, May & Stork (2013)). Yet the Catalogue of Life website stated on 16 September 2020 that it contained listings of 1,854,034 living species, thought to be “probably just over 80%” of the 2.2 million species currently known to taxonomists. These estimates, based on the same underlying data, differ by 30%. The Catalogue of Life website no longer estimates completeness, but as of 05 September 2021 it stated that their database contained 2,016,676 species (https://www.catalogueoflife.org/data/taxon/5T6MX), of which 77,977 were “provisionally accepted” (as stated in the website’s search interface).

We use terrestrial Mollusca as a testbed for developing tools for estimating known species richness. All terrestrial mollusks are gastropods; see Box 1 for a summary of their terminology, classification and habitats. Mollusca is the second most species-rich phylum after the Arthropoda, so techniques that succeed in estimating molluscan diversity should be applicable to overall eukaryote diversity. Better estimates for molluscan species richness in particular are sorely needed as the Mollusca have been hardest hit by historical extinction among all groups of organisms (Lydeard et al., 2004; Régnier, Fontaine & Bouchet, 2009). Régnier et al. (2015) estimated that 7–10% of Recent (stylommatophoran) land snail species are already extinct, yet we do not know how many land snail species have already been described. Rosenberg (2014) estimated that there are about 24,500 described species of Recent terrestrial mollusks, similar to the Lydeard et al. (2004) estimate of 24,000 species, but MolluscaBase (MolluscaBase, 2021) now exceeds those totals for accepted species of land snails, with more than 27,000 as of July 2021, and is not yet complete. Better estimators of known species richness are needed that allow uncertainty of estimates to be quantified.

**Box 1:** About mollusks. All terrestrial mollusks are gastropods, but terrestrial gastropods are not monophyletic; we summarize here their classification as used in MolluscaBase.

The Recent Gastropoda are divided into six subclasses: Patellogastropoda, Vetigastropoda, Neomphaliones, Neritimorpha, Caenogastropoda and Heterobranchia (Bouchet et al., 2017; MolluscaBase, 2021). The first three are marine only; the other three have marine, freshwater and terrestrial members. Subsets of terrestrial gastropods are often referred to as “pulmonates” (with a lung rather than a gill for gas exchange) and “operculates” (with a horny or calcareous operculum to close the shell aperture), which are grades, not clades. All known terrestrial heterobranchs are hermaphrodites, and, with a single exception, all terrestrial heterobranchs are members of Eupulmonata. The exception is an acochlidiimorph slug; all other slugs are members of either Stylommatophora, which contains most land snails and is entirely terrestrial, or Systellommatophora. All systellommatophorans are slugs, some of which are marine. The Ellobiida are also eupulmonates; they are shell-bearing and are either marine or terrestrial. The operculates include the neritimorph and caenogastropod land snails; most are dioecious, but a few can reproduce parthenogenetically. Among Neritimorpha, the superfamilies Helicinoidea and Hydrocenoidea are entirely terrestrial and there are also a few terrestrial species in Neritoidea. Terrestrial caenogastropods are either cyclophoroids, all of which are terrestrial, or littorinimorphs, which are variably terrestrial, freshwater, or marine.
***Classification of Gastropoda, showing clades with Recent terrestrial members***
Subclass Neritimorpha
Order Cycloneritida
Superfamily Helicinoidea
Superfamily Hydrocenoidea
Superfamily Neritoidea
Subclass Caenogastropoda
Order Architaenioglossa
Superfamily Cyclophoroidea
Order Littorinimorpha
Superfamily Littorinoidea
Superfamily Truncatelloidea
Subclass Heterobranchia
Infraclass Euthyneura
Subterclass Tectipleura
Superorder Acochlidiimorpha
Superfamily Acochlidioidea
Superorder Eupulmonata
Order Ellobiida
Order Stylommatophora
Order Systellommatophora

We expand here on the methods of Rosenberg (2014), who estimated the number of named marine mollusks as 43,600 ± 900 species using the binomial distribution to obtain error bars (standard deviations) for random sampling of the World Register of Marine Species (the parent of MolluscaBase), compared to the collection database for malacology at the Academy of Natural Sciences of Philadelphia (Drexel University). Rosenberg (2014) did not note that his procedures were analogous to mark-recapture methods. Estimation of the number of species named by a certain date has the same mathematical underpinnings as the Lincoln-Petersen estimator for mark-recapture (Petersen, 1896; Lincoln, 1930; Seber, 1982), but a different set of assumptions must be met. One of the key assumptions is that the databases used in the estimate are independent and uncorrelated, in this case, MolluscaBase, and the collection database for Recent Mollusca at the Academy of Natural Sciences of Philadelphia (ANSP). These databases are independent for terrestrial mollusks—that is, they were compiled independently. MolluscaBase was not used in the curation of terrestrial Mollusca in the ANSP collection and the collection database had not before this exercise been queried for names of terrestrial mollusks missing from MolluscaBase. ANSP has long pursued having a collection as complete as possible for molluscan species and incorporates collections of many individuals that specialized in land snails, among them Robert Swift (Clench, 1938), Albert Dod Brown (Foote, 1887), George Washington Tryon (Tryon, 1862; Leidy, 1867), Paul Hesse (Borrero & Rosenberg, 2015), Jens and Christa Hemmen (Kittel, Groh & Bank, 2012), and Hideo Katori (Rosenberg & Khoo, 2018). The collection holds representatives of more than half of the known species of land snails (Rosenberg, 2014).

It is desirable that the first and second samples in a mark-recapture experiment be captured with different methods to avoid correlations (Chao, Pan & Chiang, 2008). MolluscaBase and the ANSP malacology database were compiled with different methods, so they are independent methodologically. Their independence, however, does not mean that they are not correlated (Chao, Pan & Chiang, 2008). If, for example, neither MolluscaBase nor ANSP had records from Madagascar, they would be positively correlated in that aspect with diversity underestimated since a geographical region was omitted. It is also possible that MolluscaBase and ANSP are negatively correlated, in which case diversity will be overestimated. Suppose MolluscaBase is complete for a region, but ANSP is weak in that area. The result would be that more tests will be done in areas where the ANSP collection is stronger, which will lead to overestimated diversity.

In practice, there will be both positive and negative correlations and we do not know if the effects will average out. We expect that as GSDs such as MolluscaBase and other large taxonomic databases become more complete, the size of the error bars on estimates of their completeness and on known diversity of the clades and groups they cover will decrease. Therefore we can use successive estimates to understand the effect of the degree of correlation. If comparison of two estimates (in this case, one from August 2020 and one from July 2021) shows that the later estimate is significantly larger, then the databases were positively correlated; if it is significantly smaller, then they were negatively correlated, and if the estimate remains roughly constant, then the positive and negative correlations have averaged out. This will help us to judge whether the later estimate should be regarded as a minimum, maximum or accurate estimate of known diversity.

**Figure 1:**
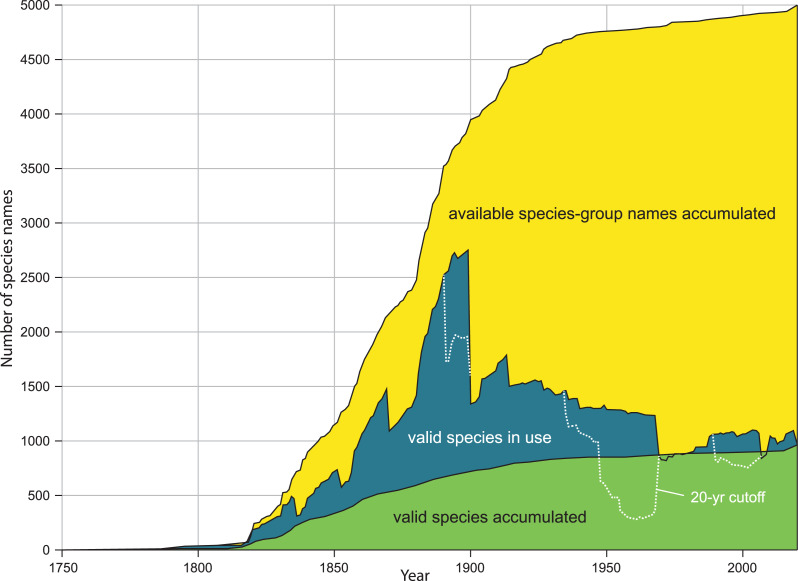
Comparison of kinds of diversity curves. Kinds of diversity curves are discussed in Box 2 and illustrated here. Yellow: accumulation of available species-group names. Green: accumulation of accepted species (species naming curve). Blue: name usage curve. Dotted line: name usage curve, with removal of any name not treated as valid in preceding 20 years. Modified from figure 2A, in Graf DL, Cummings KS. A ‘big data’ approach to global freshwater mussel diversity (Bivalvia: Unionoida), with an updated checklist of genera and species. *Journal of Molluscan Studies* 2021 87(1): eyaa034, by permission of Oxford University Press and Daniel L. Graf.

Our method estimates the height of the species naming curve (see Box 2) as of a particular date, with the 95% CI shrinking to zero as the database evaluated approaches completion. The CI is based on random sampling of the datasets that are compared. If errors or inconsistencies in the compilation of the datasets analyzed can be quantified, the error bar can be adjusted to take this additional uncertainty into account. Since our new estimate changes perception of the global diversity of land snails, we discuss the implications of this particular application of our method. The same methods can be applied to estimates of the total number of names of Recent mollusks (as opposed to names currently accepted), names of fossil mollusks, and accepted species richness in other phyla. If sufficient detail is captured, name usage curves (defined in Box 2 and shown in Fig. 1) can also be plotted from the data, which allows visualization of changing patterns of diversity in a group.

**Box 2:** Species diversity curves. Various kinds of curves showing species diversity are in use, but they have different interpretations depending on the conventions used. Following the discussion of kinds of curves, we consider implications of species concepts on perceptions of species diversity, using data from Recent birds.

***Species naming curves*** are often called species discovery curves, but that term is also used for species accumulation curves. By “species naming curve” we mean one that has year on the x-axis and number of species named by that date on the y-axis (Fig. 1). Species naming curves show a continuous increase to the current value since data are shown cumulatively. Species naming curves are generally sigmoid, with a slow initial phase, increasing slope in the middle phase of discovery, and eventually approach an asymptote reflecting that most species in the group have been named. Extrapolating total diversity from a species naming curve can be difficult unless the inventory of the group is almost complete and false plateaus are accounted for (Bebber et al. 2007). Even then, the height of the curve depends on species concepts, as discussed below.
***Name usage curves.*** Graf & Cummings (2021a) showed the distinction between a species naming curve and a name usage curve. A name usage curve shows the flux of names into and out of synonymy (Fig. 1). Alroy (2002, fig. 1) also recognized this distinction, referring to curves for “then valid” and “now valid” names. The name usage curve shows the number of names recognized on a particular date, whereas the species naming curve shows the number of names now regarded as valid that had been introduced by a particular date.
***Species accumulation curves*** rise sharply initially and eventually reach an asymptote. They are the result of sampling with a particular set of methods over a defined period of time in a restricted area. The x-axis is a measure of sampling effort, often in terms of number of individuals or samples, and the y-axis cumulates the number of species. Species accumulation curves can include morphospecies or genetic clusters rather than being restricted to entities with taxonomic names. The height of the asymptote can be calculated with rarefaction methods (Colwell, Mao & Chang, 2004; Chao et al., 2014).
***Effect of species concepts.*** For well-known groups such as birds, the species naming curve approaches an asymptote (Bebber et al., 2007: fig. 1h), as few new species are discovered each year. The name usage curve for birds, however, would show large fluctuations, with a peak of 18,937 from Sharpe (1909), who treated all subspecies as species, declining to 8,616 in Mayr (1946), but then increasing toward current values. Mayr (1946) judged that 8,616 was “probably within five per cent, and certainly within ten per cent, of the final figure”. Yet Gill, Donsker & Rasmussen (2021) recognized 11,072 species of birds, an increase of more than 28%. Of these, 10,660 had been named by 1946 and only 412 species since then, so most of the increase is from changed taxonomy, with subspecies being raised to full species.
Gill (2014) argued that the biological species concept of Mayr with its concomitant recognition of polytypic species led to the wrong null hypothesis, with reciprocally monophyletic sister populations expected to freely interbreed if they were sympatric, rather than expected not to freely interbreed. Starting with a base of 9,159 biological species, Barraclough et al. (2016) estimated that 18,043 bird species would be recognized under an evolutionary species concept (15,845 to 20,470, 95% CI) and perceived species richness would more than double to about 22,000 under a phylogenetic species concept (20,452 to 24,216, 95% CI). Bebber et al. (2007) in trying to project total species numbers for birds found that omitting most of the earliest named species gave the tightest 95% CI. Their sample included 9,961 species and yielded estimates ranging from 10,023 to 11,997 and 9,994 to 19,998 (95% CIs), depending on whether all the data or various subsets were used. Yet none of their 95% CIs reached the level predicted by Barraclough et al. under a phylogenetic species concept, which shows the importance of species concepts in determining accepted levels of diversity.

## Materials and Methods

The standard formulation of mark-recapture is as follows (Chao, Pan & Chiang, 2008). A sample n_1_ is taken from a population of size N, marked and released back into the population, giving a marked rate of n_1_/*N*. A second sample n_2_ is subsequently captured from the same population with the number previously marked being m_2_. The proportion marked in the second sample is expected to approximate the proportion marked in the population, so m_2_/n_2_ ≈ n_1_/*N*. This gives the Lincoln-Petersen estimator of population size:

(1) $${\hat{\rm N} = {\frac{n_1 \times n_2}\over{m_2}}}$$

In our study of terrestrial mollusks, we have two putatively independent samples (n_1_ and n_2_) drawn from the population of *N* named species. The samples are closed because species named after the date of sampling are excluded. The overlap between the samples is m_2_. This allows formula 1 to be used to estimate *N*. The first sample (n_1_) is the accepted names for Recent terrestrial mollusks in MolluscaBase. The second sample (n_2_) is names accepted in the collection of the Academy of Natural of Philadelphia (ANSP). Each name sampled in n_2_ has a yes or no answer to the question of whether it maps to an accepted name missing from MolluscaBase, so the distribution is binomial. Sampling is without replacement, so a hypergeometric model applies, and the variance of ${\hat{\rm N}}$ can be used to find the standard error and the 95% confidence interval (CI; formula 1.3 in Chao, Pan & Chiang, 2008).

(2) $${{\rm Var}\;(\hat{\rm N}) = {\frac{(\rm n_1\;+\;1)\;(n_2\;+\;1)\;(n_1\;-\;m_2)\;(n_2\;-\;m_2)} \over { ((\rm m_2\;+\;1)^2\; (m_2\;+\;2))}}}$$

If there are correlations between n_1_ and n_2_, then diversity might be underestimated (positive correlation) or overestimated (negative correlation) (Seber, 1982; Chao, Pan & Chiang, 2008). Potential effects from clerical, observational and other errors in addition to sampling error are considered in Supplemental Data.

In mark-recapture studies, four results are possible: an individual is in both n_1_ and n_2_, it is in n_1_ but not n_2_, it is in n_2_ but not n_1,_ or it is in neither. In estimating diversity, more results are possible since the application of a name cannot always be determined, because of *taxa inquirenda* and *nomina dubia*, therefore it is critical to consider exactly what is included in m_2_ in applying the formulas above. The number of interest is that of accepted names missing from MolluscaBase. These are the names not in m_2_, that is (1 – m_2_). Therefore, m_2_ contains all other names in the sample.

In mark-recapture studies, “equal catchability” of individuals in the population is a prerequisite for the validity of the approach (Seber, 1982; Chao, Pan & Chiang, 2008). This criterion clearly is not met for names of species. Two ways in which this problem manifests are that recently named species are likely to be included in MolluscaBase, but unlikely to be included in the ANSP collection, and that some species are more abundant or widespread than others and their names are more likely to be represented in the literature and in collections. To understand the effect of these biases, we have estimated diversity in four ways: Method (1), a “raw” estimate without any adjustments; Method (2), which excludes species first named after 2009; Method (3), which takes abundance classes into account, with classes of <5 samples, 5–15 samples, and >15 samples under that name at ANSP; and Method (4), which excludes recently named species and accounts for abundance in the ANSP collection and so is equivalent to combining Methods 2 and 3.

### Compilation of data

We present a method for estimating accepted species-level diversity, so we must clarify what we mean by “accepted” name. In zoology, accepted names are called valid names; in botany, they are called correct names (Greuter et al., 1996). To qualify as valid or correct, a name must have been published in compliance with the governing code of nomenclature. This is indeed the usage intended in the World Register of Marine Species and MolluscaBase (Horton et al., 2017). There are cases, however, where the accepted name of a taxon is not valid or correct. An example with terrestrial gastropods is *Pomatias laevigatus* (Webb & Berthelot, 1833): its original name, *Cyclostoma laevigatum*, is preoccupied by *Cyclostoma laevigatum* Menke, 1830. As a junior primary homonym, it is permanently invalid, but no replacement name has been established, so it continues to be used. It is a “current” name that is not acceptable, but it is treated as accepted in MolluscaBase. Such names are included in our counts herein.

MolluscaBase is a subset of the World Register of Marine Species (WoRMS Editorial Board, 2021) but expanded from the usual focus of WoRMS to include freshwater, terrestrial and fossil species. It is intended to be an authoritative, continuously updated GSD for Mollusca and thus provides a cornerstone of the taxonomic infrastructure for this phylum (Bieler, Mikkelsen & Giribet, 2013; Bank et al., 2014). Compilation of data in MolluscaBase is based on the standards of WoRMS as stated by Horton et al. (2017). The number of species of Recent terrestrial Mollusca was determined by using the advanced search function in MolluscaBase for Status “accepted”, Rank “Species”, Environment “Yes” for terrestrial and “Any” for other environments, and Flag “Extant, not fossil only”. Various error checks were run to find species erroneously marked as terrestrial or missing the terrestrial flag, including checking all species listed as terrestrial with at least one other environment indicated; those lacking any indication of environment; and searching for species in entirely terrestrial clades such as Stylommatophora, Cyclophoroidea, Helicinoidea or Pomatiidae that were not indicated as terrestrial.

Names used as valid in the ANSP malacology database were extracted in July 2020. Duplicates on the list were removed (*e.g*., different generic combinations of the same epithet) so that each name appeared on the list once. Each name was assigned a random number using the rand() function in Microsoft Excel. The list of 13,157 names is provided in Table S1. Our first test (completed 11 August 2020) used the first 1,100 names when sorted lowest to highest by the assigned random number. Our second test (completed 27 June 2021) used the second 1,100 names. The samples included 1,100 names so that after exclusions, more than 1,000 names would remain, ensuring large sample sizes. Names from the random samples used in 2020 and 2021 are shown in Tables S2 and S3 respectively.

Two gastropod families were set aside in the estimates: Truncatellidae and Cerionidae. Truncatellidae in ANSP and in MolluscaBase were both updated by the senior author (Rosenberg) from the same literature and so are not independent. Cerionidae are treated as nominal species rather than valid species in the ANSP collection and so do not fulfill a criterion of the test. Other reasons for exclusion were that names were determined to be out of scope (pertaining to species that are exclusively marine or fossil only), not available (*e.g*., manuscript names) or duplicates. If two different names from ANSP mapped to the same name in MolluscaBase (*i.e*., they are synonyms), only one match was counted toward m_2_ and one name was excluded, to fulfill the expectations of the binomial distribution.

Names from ANSP were assigned to taxonomic groups (Neritimorpha, Cyclophoroidea, Littorinimorpha and Eupulmonata, see Box 1) and geographic regions (Africa, Asia, Australia/New Zealand, Caribbean, Europe, North America, Oceania, South America and Other (for unassigned, *e.g*., Bermuda, or unknown)) to allow assessment of completeness in MolluscaBase for these groupings. Japan, Indonesia, New Guinea and the Philippines were grouped with Asia; Madagascar, Mauritius and the Canary Islands with Africa; the Azores and Balearic Islands with Europe; the Bahamas with North America; and the Galapagos with South America. Names that appeared in more than one geographic region were assigned to one based on type locality, area of origin (for introduced species) or preponderance of occurrences in one region in the ANSP collection. For simplicity calculations for taxonomy and geography were based on raw numbers (method 1).

## Results

**Figure 2:**
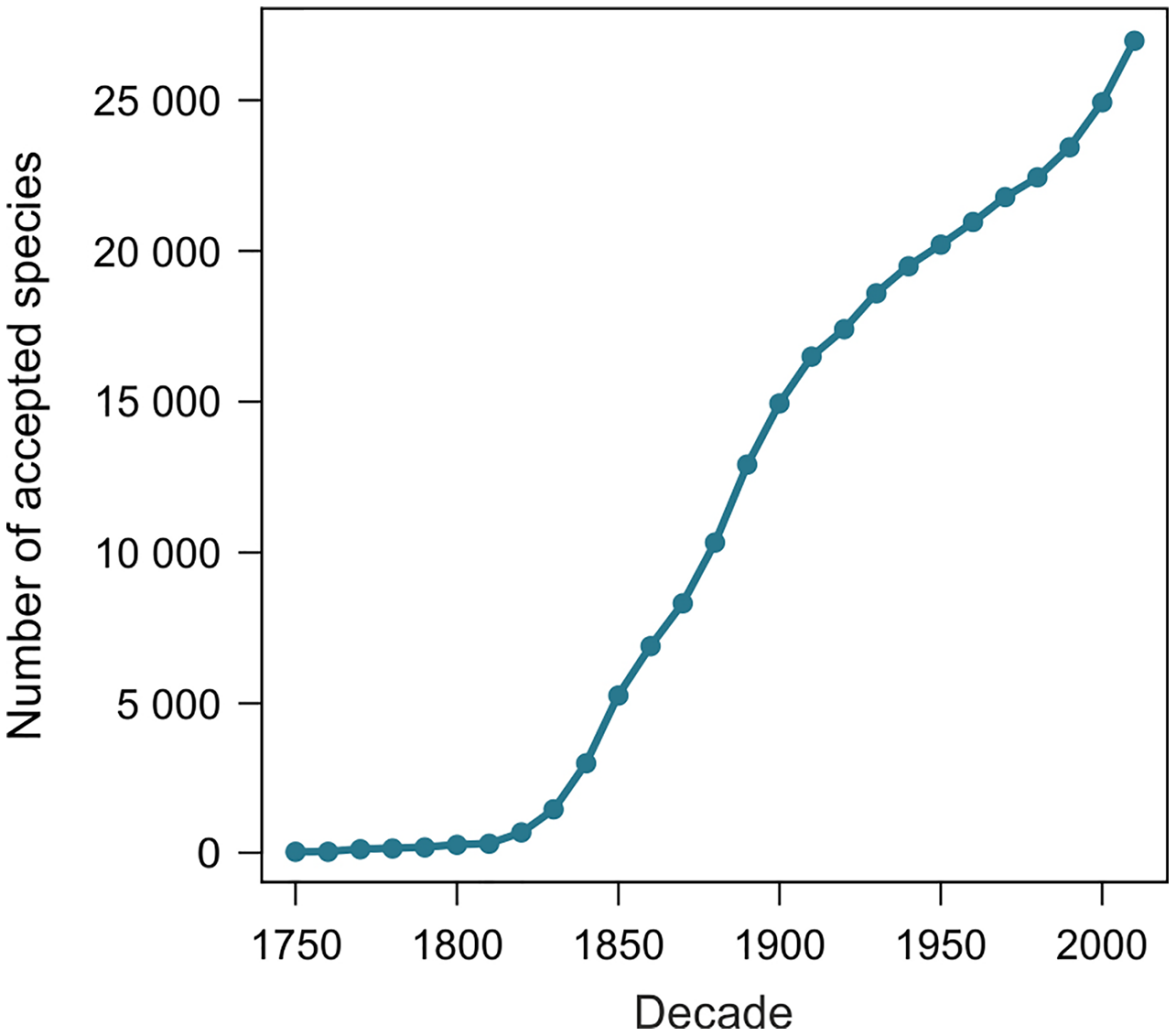
Species naming curve for terrestrial gastropods. Data are for accepted species in MolluscaBase through the end of 2020. The curve is not adjusted to account for the minor effect of replacement names, which move some names to the right.

From 11 August 2020 to 27 June 2021, the number of terrestrial mollusk species accepted in MolluscaBase rose from 24,202 to 27,050, an increase of 2,848 species. The two samples of 1,100 species yielded 1,039 and 1,036 names included in the estimates. Exclusions are summarized in Table 1 and detailed in Tables S2 and S3. The species naming curve is shown in Fig. 2 (see also Box 2 for a definition of the term).

**Table 1:** Excluded names.

Exclusion	August 2020	July 2021
Marine	1	0
Fossil only	6	2
Not available	15	11
Cerionidae	26	34
Truncatellidae	3	8
Duplicate	10	9
Total excluded	61	64
Total included (of 1,100) (n_2_)	1,039	1,036

**Note:**

Names excluded from the analyses, with reasons for exclusion. Included names comprise n_2_. See Tables S4 and S5 for geographic and taxonomic breakdowns.

Six results were possible when searching species names from ANSP in MolluscaBase: the epithet was present and the currently accepted name was present; the epithet was present but the accepted name was uncertain or unknown (*taxon inquirendum* or *nomen dubium*); the epithet was present but the accepted name was not present (*i.e*. MolluscaBase had an erroneous status); the epithet was missing but the accepted name was present (ANSP used the name of a synonym); the epithet was missing but its status was uncertain or unknown; or the epithet was missing and the accepted name was missing. Distribution of names across these categories is shown in Table 2 and results for individual names are shown in Tables S2 and S3.

**Table 2:** Results of searching for names from ANSP collection in MolluscaBase.

Category	August 2020	July 2021
Accepted names in MolluscaBase	24,202	27,050
a. Epithet present and accepted name present	818	943
b. Epithet present, accepted name uncertain or unknown	3	0
c. Epithet present, accepted name missing (MB status wrong)	5	10
d. Epithet missing but accepted name present	23	19
e. Epithet missing, accepted name uncertain or unknown	16	9
f. Epithet missing and accepted name missing	174	55
Total, a through f (n_2_)	1,039	1,036
Percent accepted names missing (line f/n_2_)	16.7%	5.3%

**Note:**

Names in MolluscaBase (MB) are in n_1_; those in the ANSP collection are in n_2_. Six results were possible, shown in lines a–f. Line f corresponds to (1 − m_2_), so lines a-e are in m_2_. Details by geography and taxonomy are shown in Tables S4 and S5.

**Figure 3:**
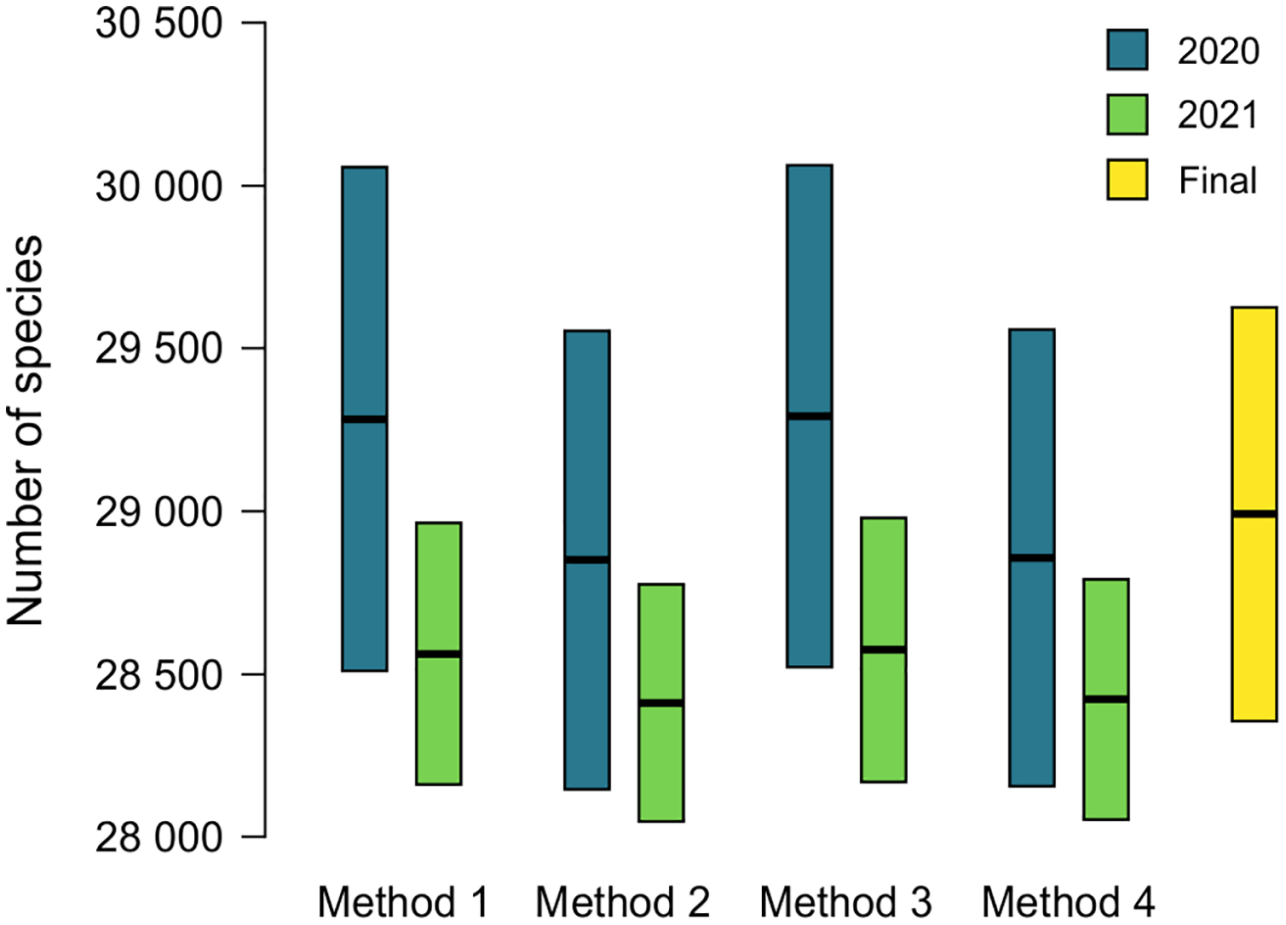
Estimates of accepted species-level diversity of terrestrial gastropods. Comparison of estimates from 2020 (blue) and 2021 (green), with 230 species added to the numbers for 2020 to make the years directly comparable (230 = 2,385 − 2,155, from the lines for recently named species in Tables 4 and 3 respectively). Method 1 uses unadjusted numbers, method 2 accounts for recently named species, method 3 accounts for “abundance” at ANSP, and method 4 accounts for both recent naming and abundance at ANSP. The fifth column (yellow) indicates our final adjusted estimate for the end of 2021 (see Table 5).

The name used as valid at ANSP will lead to the addition of an accepted name to MolluscaBase when both the epithet and the accepted name are missing. This occurred for 174 names (16.7%) in 2020 and 55 names (5.3%) in 2021 (Table 2). Estimates of diversity based on formulas 1 and 2 above are shown in Tables 3 and 4, ranging from 28,621 ± 704 to 29,063 ± 771 in 2020 and 28,409 ± 365 to 28,572 ± 406 in 2021. Comparison of estimates from 2020 and 2021 is shown in Fig. 3, with 230 species added to the total for 2020 to account for species named since the earlier estimate was completed. Estimates from 2020 are slightly higher than those for 2021, but all the estimates have overlapping 95% CIs, which indicates that positive and negative correlations between MolluscaBase and the ANSP collection averaged out. As expected, estimates from 2021 have smaller 95% CIs than those from 2020, approximately ± 400 *vs*. > ± 700.

**Table 3:** Estimates of diversity in 2020.

11 August 2020	Method 1	Method 2	Method 3				Method 4			
ANSP samples	All	All	<5	5–15	>15	Total	<5	5–15	>15	Total
n_1_	24,121	21,966	15,438	5,409	3,273	24,121	14,059	4,926	2,981	21,966
n_2_	1,039	1,039	665	233	141	1,039	665	233	141	1,039
m_2_	865	865	553	190	122	865	553	190	122	864
${\hat{\rm N}}$	28,973	26,385	18,565	6,633	3,783	28,982	16,906	6,041	3,445	26,392
95% CI	774	704	619	393	238	771	563	357	216	701
				
Truncatellidae*	56	56	56				56			
Cerionidae*	25	25	25				25			
Recently named**	N/A	2,155	N/A				2,155			
Adjusted estimate	29,054	28,621	29,063				28,628			

**Notes:**

Estimates of diversity are based on formula 1 with the 95% confidence interval calculated as sqrt (var(${\hat{\rm N}}$)) × 1.96 from var(${\hat{\rm N}}$) in formula 2. The total number of accepted species names in Mollusca on 11 August 2020 was 24,202; n_1_ uses this basis less Truncatellidae and Cerionidae and, in some methods, recently named species. Methods are: (1) raw calculation, (2) accounting for recently named species, (3) accounting for the number of occurrences of names at ANSP, and (4) accounting for factors in both 2 and 3. For methods 3 and 4, observed proportions of n_2_ were used to partition n_1_. Excluded names were added at the end to yield an adjusted estimate with the same 95% CI.

* Accepted in MolluscaBase by 11 Aug 2020, with Truncatellidae lacking the terrestrial flag excluded.

** Named since 2009 and added to MolluscaBase by 11 Aug 2020, less 7 cerionids, 8 truncatellids and 7 replacement names introduced in that period. The replacement names are excluded because the name replaced was previously listed and so is already accounted for.

**Table 4:** Estimates of diversity in 2021.

27 July 2021	Method 1	Method 2	Method 3				Method 4			
ANSP samples	All	All	<5	5–15	>15	Total	<5	5–15	>15	Total
n_1_	26,937	24,535	17,473	6,136	3,328	26,937	15,915	5,589	3,031	24,535
n_2_	1,036	1,037	672	236	128	1,036	672	236	128	1,036
m_2_	981	981	632	221	128	981	631	2221	128	981
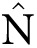 ${\hat{\rm N}}$ $\hat{N}$	28,447	25,911	18,579	6,553	3,328	28,459	16,922	5,968	3,031	25,922
95% CI	402	365	346	212	0	406	315	193	0	369
				
Truncatellidae*	66	66	66				66			
Cerionidae*	47	47	47				47			
Recently named**	N/A	2,385	N/A				2,385			
Adjusted estimate	28,560	28,409	28,572				28,420			

**Notes:**

Estimates of diversity in 2021 with methods as stated in the caption of Table 3. The total number of accepted species names in MolluscaBase on 27 July 2021 was 27,050.

* Accepted in MolluscaBase, with Truncatellidae lacking the terrestrial flag excluded.

** Named since 2009 and added to MolluscaBase by 27 Jul 2021, less 7 cerionids, 8 truncatellids and 8 replacement names introduced in that period.

The effects of potential errors and inconsistencies in compilation of the data beyond those reflected in the sampling error are estimated at 475 ± 430 species (Table 5). Adding this additional uncertainty to the estimate from method 4 in 2021 of 28,420 ± 369, which also accounts for biases in the data, yields our final estimate of terrestrial gastropod diversity of 28,895 ± 630 species known by the end of 2021 (Fig. 3) (See the caption of Table 5 for determination of the uncertainty term). We round this estimate to 29,000 at some places in the following discussion.

**Table 5:** Factors beyond sampling error.

Category	Low	High	Comment
Validity overlooked	108	514	Binomial 95% CI for 15/2075 examples
Synonymy overlooked	−170	−30	Binomial 95% CI for 6/2075 examples
Cerionidae	117	117	In addition to accepted names present 11 Aug 2021
Recently named	−200	0	Additional effect of excluding some names before 2010
Uncertain status	33	33	Status incorrectly listed as “uncertain”
Temporary status	2	2	Status incorrectly listed as “temporary”
Fossil status wrong	0	30	Listed as fossil but also extant
“Recent” status wrong	−10	0	Listed incorrectly as Recent but fossil only
Extinct in last 500 years	5	30	Listed as fossil only but survived into the last 500 years
Duplicate entries	−81	−20	Two entries instead of one for an accepted species
Named in 2021	140	140	Added to MB after 11 Aug 2021
Assimineidae	0	45	Environment terrestrial with flag “unknown”
Truncatellidae	0	16	Environment terrestrial with flag “unknown”
Ellobiidae	0	129	Environment terrestrial with flag “unknown”
Totals	−56	1,006	Average, 475

**Note:**

Factors beyond sampling error affecting the estimate of accepted diversity for terrestrial gastropods. The columns “Low” and “High” show the range of number of species that might be affected, with negative numbers reducing the estimate and positive numbers increasing it. Totals by column are shown at the bottom, with midpoint of 475 ((1,006 − 56)/2). The overall effect is 475 ± 430 species. The error bar was calculated as the square root of the sum of squares of (high − low)/2 for items expected to be random with relation to one another (240) plus the total where environment has “terrestrial” flagged as unknown (190), since these might all fall one direction, depending on definitions. The uncertainty term for the overall estimate of diversity was determined as sqrt (369^2^ + 240^2^) + 190 = 630. See Supplemental Data for further explanations.

The geographic breakdown of species missing from MolluscaBase is shown in Table S4 with raw data in Tables S2 and S3. The two biggest gaps in 2020 were in the Caribbean and Oceania, missing 57% and 41% respectively. Both areas showed substantial improvement by 2021, to 21% and 9%, but still had the largest percentages of missing species among the regions. Continental regions had lower percentages of missing species, with some areas being essentially complete (Australia/NZ, Europe, and North America). Overall, 80% of missing species were on islands in the first sample, and 75% in the second sample. Among continental regions, only Africa was higher than the average across all regions for missing species, with 17.6% in 2020 and 6.5% in 2021 *vs*. overall of 16.7% in 2020 and 5.3% in 2021.

The taxonomic breakdown of missing species is shown in Table S5 with raw data in Tables S2 and S3. The eupulmonates have a similar proportion missing to the overall dataset, partly because they are numerically dominant, constituting about 80% of all terrestrial gastropods. They had 16.4% missing in 2020, while operculates had 18.4%, but both groups improved in 2021, to 5.2 and 5.7% missing respectively. Littorinimorphs (primarily Annulariidae) had the highest proportion of missing species, 52.6% in 2020 and 16.7% in 2021. Neritimorphs improved from 22.2% missing in 2020 to 4.3% in 2021. Cyclophoroids improved from 4.0% missing in 2020 to essentially complete for accepted species in 2021.

## Discussion

### Adaptation of mark-recapture methods

Although mark-recapture methods and estimating known species diversity have similar mathematical underpinnings, they have different assumptions. Mark-recapture relies on the assumption of “equal catchability” (Seber, 1982; Chao, Pan & Chiang, 2008) but this assumption clearly is not met for estimates of known species diversity. Despite not meeting this standard, estimates of diversity seem not to be greatly affected by departures from it. One area where this bias manifests is that MolluscaBase is virtually complete for species of mollusks named in the last 10 years, since they are easily discovered with searches in Google Scholar, Zoological Record, and ZooBank, whereas ANSP and other collections are weak for these species. Although collections may contain them, they have not yet been identified therein. Since the ANSP collection and MolluscaBase are negatively correlated in this respect, diversity should be overestimated. When recently named species are set aside (method 2), estimates from 2020 decreased by 433 species (1.5%) and from 2021 by 152 species (0.5%) (Fig. 3; Tables 3–4). The magnitude of the effect is inversely correlated to completeness of the enumerations: in 2021, only 5.3% of sampled species led to missing accepted species; in 2020, the corresponding figure was 16.7% (Table 2). Since the estimates from 2021 are within the error bars for the estimates of 2020, the effect is not significant, but it could be significant in cases where the database is less complete.

A different situation is seen when taking abundance into account. When samples from ANSP are divided into three abundance classes (<5, 5–15 and >15 samples corresponding to a name), there is a negligible increase: nine species in 2020 and 12 species in 2021 (Tables 3–4). Rosenberg (2014, table 4) had a similar result with marine mollusks, with estimates increasing by about 0.5% when abundance was taken into account, but with error bars overlapping the raw estimate. Rosenberg’s (2014) procedure was different from the one used here, with stratified sampling across five abundance classes instead of three, so that each group had at least 200 randomly chosen names, but the overall conclusion is the same: abundance has little effect on the estimate of species-level diversity. This makes sense because discoverability of a name has little correlation with abundance of the organism it represents. When a species is named, it is likely picked up in several indexing sources. If it is missed by the research community, it fades from knowledge (see “overlooked names” in Supplemental Information). Organisms known from only one specimen might be cited hundreds of times and their status as accepted species debated. The dinosaur *Hadrosaurus foulkii* Leidy, 1858, for example, was considered a *nomen dubium* by Prieto-Márquez, Weishampel & Horner (2006), a conclusion later reversed on further study (Prieto-Márquez, 2011). Yet, one of the most abundant organisms in the world, the cyanobacterium *Prochlorococcus marinus*, was not named until 1992 (Chisholm et al., 1992) and many abundant species remain unnamed (Leray & Knowlton, 2016).

The methods also differ in that mark-recapture focuses on recaptured individuals, whereas our estimates of diversity focus on species that were not “recaptured”, that is, accepted names missing from MolluscaBase. Since we defined this number to be (1 − m_2_), it can readily be converted to m_2_ for use in the mark-recapture formulas. Names in m_2_ in estimates of diversity, however, should not be thought of as recaptured, because there are names where we cannot tell whether they were recaptured. These correspond to item e in Table 2: “Epithet missing, accepted name uncertain or unknown”. Names in item b in Table 2, “Epithet present, accepted name uncertain or unknown”, appear similar but are not problematic—their status is given in MolluscaBase, because the epithet is present.

The method presented herein provides a means of estimating the number of species currently accepted in a group that does not yet have a complete taxonomic database, but the 95% CIs provided are based only on sampling error. Our best estimate for accepted diversity of terrestrial gastropods in 2021, accounting for potential correlations between the databases, is 28,420 ± 369 species. Additional uncertainty comes from clerical and observational errors. We have controlled for these errors to the degree possible (see Table 5 and Supplemental Data), but they grade into taxonomic uncertainty. Adding in the additional uncertainty from clerical and observational errors of 475 ± 430 (Table 5) yields a rounded estimate of terrestrial gastropod diversity of 28,895 ± 630 species known by the end of 2021 (Fig. 3). The added uncertainty means that the error term represents a range rather than a statistical confidence interval.

Taxonomic databases contain “eclectic species” (Blackburn & Tyler, 1987; Barraclough et al., 2016), discovered with various methods and proposed under a variety of taxonomic traditions and species concepts. Technological advances such as scanning electron microscopy, micro-CT scanning and DNA sequencing mean that the methods used by taxonomists are changing—more data sources are available and methods of analysis grow more powerful. Integrative methods lead to better understanding of taxa (Pante, Schoelinck & Puillandre, 2015; Goulding & Dayrat, 2016), as have debates about species concepts (Padial & De la Riva, 2006). Since it is not possible to revise all the species in large groups simultaneously, current methods will not yet have been applied to some of the component taxa (Gaston, 2008). Also, some species concepts may not be appropriate for various groups—Mollusca for example contains some groups that are parthenogenetic, so their members cannot be treated under the Biological Species Concept. Large taxonomic databases thus will always contain eclectic species. (See Box 2 for an example of the importance of species concepts).

Our method estimates the height of the species naming curve (Box 2), with the error bar shrinking to zero as the database evaluated approaches completion. A species naming curve may show known diversity approaching an asymptote, as with unionoids (Fig. 1), or rapidly increasing as with marine mollusks (Rosenberg, 2014, fig. 1), terrestrial gastropods (Fig. 2), and many other groups of organisms (Bebber et al., 2007; Edie, Smits & Jablonski, 2017). As taxonomic research progresses, the number of species recognized in a group as of a given date may increase or decrease, depending on the flux of names into and out of synonymy. This flux can be shown in a name usage curve (Fig. 1). Alroy (2002), based on North American fossil mammals, proposed a method to take this flux into account in correcting estimates of accepted diversity, but MolluscaBase does not have the detail necessary to implement his method. It might be implemented for freshwater mussels (Unionida) since Graf & Cummings (2021a; 2021b) have captured all taxonomic actions relevant to the group in the MusselP database.

In principle, the range of current values determined by our method could be incorporated into projections of diversity levels that might ultimately be recognized. A rapidly increasing curve like that for terrestrial gastropods indicates that many species remain to be discovered, but trying to extrapolate an upper limit is likely to be futile since the error bars become very large (Bebber et al., 2007; Gaston, 2008). Estimates are even less reliable as additional assumptions are made. Appeltans et al. (2012), for instance, incorporated synonymy rates, rates of discovery, and projected tens of thousands of cryptic species yet to be discovered in trying to estimate the scale of global marine diversity. They concluded that previous estimates greatly exceeding one million marine species were “highly unlikely”. Yet, Leray & Knowlton (2016) gave credence to estimates greater than one million because DNA-based approaches show many more lineages than projected by Appeltans et al. (2012). According to Fišer, Robinson & Malard (2018), the increasing ability to detect cryptic species and the history of lineages constitutes a paradigm shift. Korshunova et al. (2019) have argued, however, that increasing ability to make fine scale morphological and molecular distinctions means that degree of crypticity is a continuum, so that it is misleading to refer to cryptic species. These views are concordant, however, in illustrating how increasing technological abilities and theoretical understanding increase our ability to detect species, but they also make it harder to predict an endpoint.

### Global diversity of recent terrestrial gastropods

Most mollusk species are still discovered and described under a morphological species concept, although many groups of mollusks exhibit non-adaptive or morphostatic radiation (Gittenberger, 1991; Davis, 1993; Falniowski, 2018). “Cryptic” or morphostatic species have been documented in marine (Meyer, Geller & Paulay, 2005; Fassio et al., 2020), freshwater (Wilke et al., 2010; Falniowski et al., 2020) and terrestrial mollusks (Köhler & Burghardt, 2016; Moussalli & Herbert, 2016; Liew et al., 2014). Descriptions of new species and subspecies of mollusks often do not define their taxonomic concepts (Rosenberg, Moretzsohn & García, 2009) and different schools of thought may have different underlying concepts of taxa (Rosenberg & Ludyanskiy, 1994; Graf, 2007). Molluscan subspecies can be particularly problematic. Many named prior to the geographic subspecies concept (Mayr, 1942; Wilson & Brown, 1953) were sympatric with the nominate subspecies. Modern authors encountering subspecies that are sympatric either synonymize them if they intergrade or treat them as distinct species if they do not (*e.g*., Rosenberg & Muratov, 2006; Mason et al., 2020). Rosenberg & Muratov (2006) provided guidelines for evaluating older names, since many of the taxa they treated in compiling the Jamaican terrestrial fauna had not been considered since the 1930s or before. Names are taxonomic hypotheses that can be reevaluated as needed when a researcher considers whether a name already exists for a taxon (Gittenberger, 1993; Rosenberg, 1993). A taxonomic hypothesis that has been tested and supported provides a better basis for decision making than one that has not been (Gaston & Mound, 1993). (See Supplemental Data under “species concepts” for examples with *Cerion.)*

Although many previously named molluscan species-group taxa have not been evaluated with modern methods, it is nonetheless possible to predict on a qualitative basis some of the effects of taxonomic revision on taxa that have already been named. As of 16 Aug 2021, MolluscaBase had 2,300 *taxa inquirenda*, 6,400 subspecies and more than 10,000 synonyms for terrestrial gastropods. MolluscaBase is not as complete in its treatment of synonyms and subspecies as it is for accepted species, but these numbers show that there is considerable potential to add accepted species by clarifying *taxa inquirenda* and reevaluating synonyms and subspecies. That means the species naming curve (Fig. 2) will increase in height, even without new discovery, since resurrected taxa enter the curve in the year they were named. On a name usage curve (Box 2) in contrast, a name would be counted from when it was named to when it was first synonymized and then reappear in the count in any subsequent interval when it was accepted.

The shape of the species naming curve shows that discovery of terrestrial gastropods is far from complete (Fig. 2), as is true for many other taxa (Bebber et al., 2007; Edie, Smits & Jablonski, 2017). Many mollusks were originally described from shells alone. When modern methods are applied integrating data from shell morphology, anatomy and molecules (Goulding & Dayrat, 2016) new species are often discovered (*e.g*., Criscione & Köhler, 2013). Solem (1984) predicted that the median range for all land snail species would be “less than 100 km, and quite probably less than 50 km”, which suggests that some regions have not been sampled with sufficient intensity to discover most of their species. On average, more than 200 species of terrestrial gastropods have been named per year since 2009, with rates of discovery currently accelerating. Both revisions of existing names and the trajectory of discovery are pushing the species naming curve higher. Although it is not currently possible to predict an upper limit to accepted species level diversity of terrestrial gastropods, it is clear that our estimate of 29,000 species already accepted is a minimum. It would not be surprising to see accepted diversity reach 50,000 species or more.

Solem (1984) estimated the actual minimum diversity of land snails including species yet to be named as 30,000 to 35,000. The number of accepted species is now approaching that lower bound. Accepted names for Europe, North America and Australia/New Zealand are essentially complete. Among continental areas, Africa had the largest percentage missing, but all of the missing taxa are from North Africa, indicating that accepted names from sub-Saharan Africa are virtually complete. The largest remaining gaps were with island faunas. In the Caribbean the fauna of Cuba and in Oceania that of the Hawaiian Islands are the most diverse and these also account for many of the species missing from MolluscaBase. Completeness for accepted names in MolluscaBase does not indicate that a region is well known, since it may be many years since its fauna was treated.

Recent non-marine mollusks have among the highest extinction rates of any group of organisms (Lydeard et al., 2004; Régnier, Fontaine & Bouchet, 2009). Régnier et al. (2015) estimated that 7–10% of Recent terrestrial gastropods species have gone extinct during the Anthropocene. Small geographic ranges put species at immediate risk of extinction. The criteria for evaluation as endangered on the IUCN Red List include extent of occurrence less than 5,000 km^2^ or area of occupancy less than 500 km^2^. If Solem (1984) is correct in suggesting that the median range size of land snail species is less than 50 km, then more than half of the species fulfill an area criterion for endangered status.

### Outlook

Our adaptation of mark-recapture methods to estimating accepted species diversity is applicable to taxonomic databases for other phyla and will enable more sophisticated tests of the completeness of global species databases (GSDs). If our method is applied to paleontological databases, a stratified sampling approach should be used to ensure adequate coverage across geological epochs. If appropriate versions or backups of a database exist, it may be possible to implement our method comparing present to past versions instead of needing to wait for database growth. The result will be better understanding of the completeness of existing GSDs.

As taxonomic coverage in GSDs increases, it will no longer be necessary for researchers to compile their own “graveyards” of names (Gittenberger, 1993) to work on a taxon. As geographic coverage increases, it will be possible to generate faunal lists for regions that lack them. As images of type specimens accumulate it becomes possible to more rapidly access identities of nominal taxa. This will lead to acceleration of research in systematic biology, evolution, ecology and conservation.

## Conclusions

Mark-recapture methods can be successfully adapted to estimating accepted species level diversity from comparisons of independent taxonomic databases. The estimate allows a confidence interval to be placed on the height of a species naming curve.

In the cases of terrestrial gastropods all estimates from two samples a year apart had overlapping 95% CIs, indicating that correlations between the databases did not cause significant problems. Taking abundance into account did not have a significant effect on the estimates. Taking into account other sources of uncertainty, we estimate accepted species-level diversity of terrestrial gastropods as 29,000 species (28,895 ± 630) at the end of 2021. This is a minimum estimate of true diversity. Attempts to project future levels of species richness that might be recognized must take into account both revision of known taxa and discovery of new species.

The methods presented here can be applied to estimate the total number of names of Recent mollusks (as opposed to names currently accepted), the diversity of fossil mollusks, and diversity in other phyla.

## Supplemental Information

10.7717/peerj.13139/supp-1 Supplemental Information 1 Supplementary data. Various kinds of errors can affect the reliability of estimates of named diversity. These are discussed in this section, and the sizes of expected effects on the current estimate are summarized in Table 5. The effect of species concepts is also considered.

10.7717/peerj.13139/supp-2 Supplemental Information 2 Complete list of land snail names at ANSP. Each line represents one species of land snail regarded as valid in the ANSP collection in August 2021. The family assignment at ANSP and the random number assigned to each name are also shown.

10.7717/peerj.13139/supp-3 Supplemental Information 3 Random sample of names from ANSP in 2020 and results of matching to names in MolluscaBase. Each row represents a name treated as valid in the first random sample from ANSP. Columns are: “Sort”, showing 1–1100, assigned by sorting low to high by the next column “Random”, a random number assigned in Excel. “Binomen” is the name sampled, excluding the subgenus name if any from the next column, “Full name”. “"ANSP authority” shows the authorship attribution at ANSP and "“Family” shows the family name used at ANSP. “Total lots” shows how many samples are presented in the ANSP collection. “Abundance” codes are “l” [low] for 1–4 lots, “m” [medium] for 5–15 and “h” [high] for >15. “Aphia ID” is the unique identifier from WoRMS/MolluscaBase. “Match type” show the results of automated matching supplemented by manual searches for epithets. “Taxon status” shows our judgement of the status of the name. “ScientificName” and “Authority” correspond to the Aphia ID. “Aphia ID accepted”, “Species_accepted” and “Authority accepted” are the accepted data in MolluscaBase for the matched names. “Main Region” shows the continental or oceanic area for a taxon (summarized in Table S4) and “Group” gives the main taxonomic groups: “N”, Neritimorpha, C, Cyclophoridea, L, Littorinimorpha, and P, Eupulmonata (summarized in Table S5). “Available” shows if a name was properly published and so can be used for a taxon; “Out of scope” flags names that are fossil only or marine; “Duplicate output” flags accepted names matched more than once; “Exclude” flags names excluded from the analysis on the basis of the three preceding columns. “Epithet missing” and “Accepted name missing” provide the 6 categories in Table 3: with “n”, “y” or “??” possible in both columns. “Comment” summarizes the results of our research into the status of these names. Initial sort order is by “Exclude”, “Epithet missing”, “Accepted name missing” and “Group” in that order.

10.7717/peerj.13139/supp-4 Supplemental Information 4 Random sample of names from ANSP in 2021 and results of matching to names in MolluscaBase. Each row represents a name treated as valid in the second random sample from ANSP. Columns and their descriptions are the same as in Table S2, except that “Sort”, shows numbers 1101–2200, following 1–1100 in Table S2.

10.7717/peerj.13139/supp-5 Supplemental Information 5 Geographic summary. Geographic distribution of names sampled at ANSP, showing percent accepted names missing from MolluscaBase by region. Raw data are in Tables S2 and S3.

10.7717/peerj.13139/supp-6 Supplemental Information 6 Taxonomic distribution. Taxonomic distribution of names sampled at ANSP, showing percent accepted names missing from MolluscaBase by clade (or grade, *i.e*., operculates). Raw data are in Tables S2 and S3.

## Additional Information and Declarations

### Data Availability

The following information was supplied regarding data availability: The raw data, geographic and taxonomic summaries of the data are available in the Supplemental Files.
