# Supplementary material for: Adapting mark-recapture methods to estimating accepted species-level diversity: a case study with terrestrial Gastropoda
Source: PeerJ. 2022 Jun 21;10:e13139. doi: 10.7717/peerj.13139 (PMC9231345; doi:10.7717/peerj.13139)
Supplement: Supplemental Information 1 — Various kinds of errors can affect the reliability of estimates of named diversity. These are discussed in this section, and the sizes of expected effects on the current estimate are summarized in Table 5. The effect of species concepts is also considered. [file peerj-10-13139-s001.docx]

**SUPPLEMENTAL DATA**

**Incorrect status of names in MolluscaBase**

It is up to the individual MolluscaBase editor’s discretion whether a name with no recent treatment is accepted or treated as a *taxon inquirendum*. These are conflicting actions, however, since one puts a name in m_2_, the other excludes it. Horton et al. (2017) did not give a precise cut-off for what “recent treatment” means. In Rosenberg (2014) names of marine mollusks not treated since 1950 were categorized as uncertain or unknown. But with terrestrial mollusks, many groups and regions have had no revision since 1950. Using “taxon inquirendum” as a placeholder, meaning that the name has not been researched, however, leads to bias when the name should have been accepted. Such names were discovered by our random sampling (3 in 2020 and 6 in 2021) and are included in item c of Table 3.

According to Horton et al. (2017) the correct way to indicate such names is with status “uncertain” and label “unassessed” [in the field for “Unacceptreason”]. “Uncertain” *has* been used in this sense, but often without the label. Names that correctly have this status usually lack author and date. The status “uncertain” or “temporary name” has also been incorrectly used when “incertae sedis” is meant. Horton et al. (2017) said that each *taxon inquirendum* should have a note explaining its status, but less than 4% of almost 8,000 *taxa inquirenda* in the species group in MolluscaBase have such a note. Reviewing the status of these names would likely find more names that should be accepted.

We also found a few names treated as synonyms that should have been treated as accepted: 2 in 2020 and 4 in 2021. These are also included in item c of Table 3. We have counted all names from item c in m_2_ since the problem is not that the names are missing from MolluscBase, but that they have the wrong status. That is, the error is part of uncertainty beyond that from sampling error. If we had treated them as additional missing names, the estimate for 2020 would increase by 119 to 132 species and for 2021 by 267 to 298 species. In Table 5 we added together items from 2020 and 2021 that had incorrect status (15 out of 2075) and used the binomial confidence interval based on 27,050 names to find high and low values for the effect. We used  [statpages.info/confint.html#Binomial](https://statpages.info/confint.html#Binomial) to help calculate CIs.

Although we can demonstrate that there are names treated as synonyms that should have been treated as accepted, we do not know the size of the opposite bias: names accepted in MolluscaBase that should not be. Determining a value for this bias quantitatively would require random sampling of names from MolluscaBase rather than from the ANSP collection. It is likely that an incorrect status of “taxon inquirendum” or “unaccepted” occurs more often than an incorrect one of “accepted”, since the taxon inquirendum has sometimes been used as a placeholder. If we set aside taxa inquirenda and assume that incorrect status of “accepted” approximately balances incorrect status of unaccepted, 6 out of 2075 names gives a CI of 30-170, which range is used in Table 5.

**Effect of setting aside recently named species**

Tables 3 and 4 show the effect of setting aside species named after 2009. Setting aside a larger set of names (e.g., those introduced after 1999) was not feasible since the random samples from ANSP contained names introduced as late as 2008, including some that were missing from MolluscaBase, so n_2_ and m_2_ would be affected in addition to n_1_. There may be a small remaining bias making the estimate too large, but it cannot easily be accounted for. We have assumed it is on the order of 0-200 species in Table 5.

**Effect of setting aside Cerionidae**

Cerionidae were excluded because they do not meet the requirements of the test, since, uniquely among the Mollusca, the ANSP collection does not distinguish valid species of *Cerion*. (The other Recent genus in the family, *Mexistrophia*, has only one species.) This is because there are no lists of accepted species in *Cerion*, which reflects that the literature has not reached consensus on *Cerion* diversity (Richardson, 1992; Harasewych, 2012). Clench (1957) listed 549 available species group names for *Cerion* [our count] but thought that less than 20% represented valid species or subspecies. Woodruff (1978) expected “a downward revision of close to two orders of magnitude” among “600 ‘species’”, and Woodruff and Gould (1980) estimated the needed reduction to be “more than an order of magnitude”. Table 1 in Woodruff and Gould (1980) presents their estimate of living diversity. Interpreting “?<30” in that table for species from Cuba as 15 to 29 and assuming that Bahamian species do not overlap among their line items we find a minimum of 64 species and a maximum of 88. The reduction in perceived diversity predicted by Woodruff & Gould (1980) is thus less than an order of magnitude, but still substantial— 6 to 8.5 times, and in at least one case is more extreme: Gould and Woodruff (1986) recognized only two species among 90 names for taxa on New Providence.

We added to the estimates the number of species of Cerionidae in MolluscaBase at the time the estimates were done (Tables 3 and 4). Since then we have prioritized data entry to Cerionidae, ending up with 164 species currently accepted, 139 and 117 more than in the estimates from 2020 and 2021 respectively. The remaining uncertainty with Cerionidae is thus pushed into other categories and is accounted for there (Table 5): either accepted status has been overlooked in the case of taxa inquirenda and synonyms, or synonymy has been overlooked in the case of accepted names.

**Effect of error or uncertainty in determining environment**

Various queries were run in MolluscaBase to find species erroneously coded as terrestrial when they were marine or freshwater, or that were terrestrial but not coded as such. Three families, however, have species where it can be difficult to tell whether they should be regarded as terrestrial: Assimineidae, Truncatellidae and Ellobiidae. Each of these groups has species that can live above the high tide line or along river banks, and in each group some species are known to be entirely terrestrial, no longer laying their eggs in water. For that reason, 190 species in these groups have “unknown” scored for terrestrial environment. Whether all, some, or none of the species end up with the terrestrial score may depend on the conventions adopted for determining terrestrial status in these taxa (see Horton et al., 2017).

**Effect of error of uncertainty of fossil status**

Some species are first named as Quaternary fossils and later found living. If the report as living is overlooked in MolluscaBase, the species will be excluded from counts when it should not have been. Such species first found living after 2021 would be added to the counts in the year named in a species naming curve, but in the year reported living in a name usage curve (Figure 2). There are also Holocene species where it is not known if they went extinct in historical times (the last 500 years) and so should be included rather than excluded from the counts herein, for example some *Hemicycla* in the Canary Islands (Beck & Rähle, 2006). There might also be species in MolluscaBase or in the random samples from the ANSP collection scored as living that should have been scored as fossil only. Lastly, there can be taxonomic disagreement whether the fossil form and the living one represent the same species.

**Duplicate entries in MolluscaBase**

Only one duplicate entry was found in MolluscaBase during our random sampling (out of 2,075), however, remaining duplicates in MolluscaBase are non-randomly distributed. They are more likely to occur in families that have many species, as they are harder to spot. A search in Camaenidae, the largest family of stylommatophorans, found 8 duplicates out of 2,668 accepted species names, which projects to 81 duplicates in 27,050 names. By “duplicate” we mean the same epithet was accepted twice at the species level, usually because it was placed in different genera. Some duplicates were also found between subspecies and species, but these do not affect the estimate. We used the projection from Camaenidae for the maximum number of duplicates. For the minimum we used (1/2075) * (27050 - 2668) + duplicates in Camaenidae = 20.

**Named in 2021**

From the time the estimate was completed on 11 August 2021, 140 species named in 2021 were added to MolluscaBase. Some recently named taxa will likely be synonymized, but taxonomic revisions that occur after 2021 are excluded from the current estimate.

**Species concepts**

The items discussed above and summarized in Table 5 have immediate effect on estimates of named diversity. Changing species concepts, however, can lead estimates of the number of species recognized as of a particular date to deteriorate over time. If in 50 years we determine how many of the names of terrestrial gastropods introduced by 2021 were accepted at species rank, the number might differ substantially from our estimate herein. With birds, estimates of species level diversity might double if a phylogenetic species concept rather than the biological species concept were applied (Barraclough et al. 2016) (see Box 2).

The importance of species concepts is also seen in works on *Cerion* (Gastropoda, Stylommatophora, Cerionidae). Gould & Woodruff (1990) said, “*Cerion* provides a classic example of a syngameon—a superspecies in which component semispecies will hybridize, often without losing their integrity”. This creates problems in a system like MolluscaBase, where “semispecies” and “superspecies” are not recognized in the taxonomic hierarchy so semispecies are mapped to species since their names have the form of species name. This equivalence forced by the database system, however, is not far from current practice, where there is increasing recognition that ability of two taxa to hybridize does not preclude recognizing them as distinct species (Mallet 2013), a possibility that Galler & Gould (1979) entertained with *Cerion*. Gould and Woodruff (1990) stated that “evolutionary species concepts based on isolation or recognition” did not work with *Cerion* but that “the evolutionary cohesion species concept introduced by Templeton (1989) is well suited to deal with this genus”.

Clearly *Cerion* forced Gould and Woodruff to grapple with species concepts, as they supported one that had not been proposed when they began their work on the group. But if they could revisit *Cerion* now, 30 to 40 years later, would they reach the same conclusions? As an example, Gould and Paull (1977) synonymized 6 taxa from Hispaniola, Mona, Puerto Rico and the Virgin Islands under *Cerion striatellum* on the grounds that they formed a morphological cline, yet their statistical analysis showed that populations on each island could be distinguished from each other. Under a phylogenetic species concept, such populations might be regarded as distinct species, which can now be tested with DNA data from tissue dried in museum specimens in a framework integrating morphological and molecular data. Such reevaluation can be important for species conservation (Prie et al., 2012). If Gould & Paull’s (1977) hypothesis were corroborated, that would provide support for there being a single widespread species of *Cerion* in the eastern part of the genus’s range. But if subspecies or full species status were supported on different islands, then perception of extinction risk would increase. Although *Cerion* usually occurs at high population density, its populations are still at risk of eradication on small islands (Shipman, 2011; Rosenberg, 2012).

Gould and Woodruff (1986) regarded themselves as clearing away a “thicket of names” that obscured the underlying biological patterns (Harasewych et al., 2007). They found in *Cerion gubernatorium* “four geographically separated subgroups”. These subgroups could have been ranked “as subspecies under [their] new taxonomic proposals”, yet they declined to recognize them with formal names. They noted that one group was extinct and “another is becoming less and less common as the city of Nassau spreads eastward”, so the threat of extinction is real.

**References cited**

Barraclough GF, Cracraft J, Klicka J, Zink RM. 2016. How many kinds of birds are there and why

does it matter? *PLOS ONE* 11(11):e0166307 DOI 10.1371/journal.pone.0166307.

Beck T, Rähle D. 2006. Description of a newly discovered extinct representative of the genus

*Hemicycla* Swainson, 1840 (Gastropoda, Pulmonata, Helicidae) from La Gomera, Canary

Islands. *Basteria* 70:53–56. [natuurtijdschriften.nl/pub/597312](https://natuurtijdschriften.nl/pub/597312).

Clench WJ. 1957. A catalogue of the Cerionidae (Mollusca: Pulmonata). *Bulletin of the Museum of*

*Comparative Zoology* 116(2):121–169. [www.biodiversitylibrary.org/page/2789645](http://www.biodiversitylibrary.org/page/2789645).

Galler L, Gould SJ. 1979. The morphology of a ‘hybrid zone’ in *Cerion*: variation, clines, and an

ontogenetic relationship between two ‘species’ in Cuba. *Evolution* 33(2):714–727

DOI 10.1111/j.1558-5646.1979.tb04723.x.

Gould SJ, Paull C. 1977. Natural history of Cerion. VII. Geographic variation of Cerion (Mollusca:

Pulmonata) from the eastern end of its range (Hispaniola to the Virgin Islands): coherent

patterns and taxonomic simplification. *Breviora* 445:1–24. [www.biodiversitylibrary.org/page/3189744](http://www.biodiversitylibrary.org/page/3189744).

Gould SJ, Woodruff DS. 1986. Evolution and systematics of Cerion (Mollusca: Pulmonata) on new

providence Island: a radical revision. *Bulletin of the American Museum of Natural History*

182(4):389–490. [digitallibrary.amnh.org/handle/2246/579](https://digitallibrary.amnh.org/handle/2246/579).

Gould SJ, Woodruff DS. 1990. History as a cause of area effects: an illustration from Cerion on

Great Inagua, Bahamas. *Biological Journal of the Linnean Society* 40(1):67–98

DOI 10.1111/j.1095-8312.1990.tb00535.x.

Harasewych MG. 2012. The fossil record and phylogeography of the family Cerionidae

(Gastropoda: Pulmonata), with the description of a new species from the Pleistocene of Florida.

*Nautilus* 126(4):119–126. [www.biodiversitylibrary.org/page/50437379](http://www.biodiversitylibrary.org/page/50437379) .

Harasewych MG, Baldinger AJ, Villacampa Y, Greenhall P. 2007. The *Cerion* (Mollusca:

Gastropoda: Pulmonata: Cerionidae) taxa of Charles Johnson Maynard and their type

specimens. *Bulletin of the Museum of Comparative Zoology* 158(7):367–523

DOI 10.3099/0027-4100(2007)158[367: TCMGPC]2.0.CO;2.

Horton TI, Gofas S, Kroh A, Poore GCB, Read G, Rosenberg G, Stöhr S, Bailly N,

Boury-Esnault N, Brandão SN, Costello MJ, Decock W, Dekeyzer S, Hernandez F, Mees J,

Paulay G, Vandepitte L, Vanhoorne B, Vranken S. 2017. Improving nomenclatural

consistency: a decade of experience in the world register of marine species. *European Journal of*

*Taxonomy* 389:1–24 DOI 10.5852/ejt.2017.389.

Mallet J. 2013. Subspecies, semispecies, superspecies. In: Levin SE, ed. *Encyclopedia of Biodiversity*.

Vol. 7. Second Edition. Waltham, Mass: Academic Press, 45–48.

Prie V, Puillandre N, Bouchet P. 2012. Bad taxonomy can kill: molecular reevaluation of Unio

mancus Lamarck, 1819 (Bivalvia : Unionidae) and its accepted subspecies. *Knowledge and*

*Management of Aquatic Ecosystems* 405(8):1–18 DOI 10.1051/kmae/2012014.

Richardson CL. 1992. Cerionidae: catalog of species. *Tryonia* 25:1-121. www.biodiversitylibrary.org/page/55547212.

Rosenberg G. 2012. Snails in peril. *American Scientist* 100(2):99.

Rosenberg G. 2014. A new critical estimate of named species-level diversity of the recent Mollusca.

*American Malacological Bulletin* 32(2):308–322 DOI 10.4003/006.032.0204.

Shipman PL. 2011. The rarest snail in the world. *American Scientist* 99(6):454–457

DOI 10.1511/2011.93.454.

Templeton AR. 1989. The meaning of species and speciation: a genetic perspective. In: Otte D,

Endler J, eds. *Speciation and Adaptation*. Sunderland, Massachusetts: Sinauer, 1–26.

Woodruff DS. 1978. Evolution and adaptive radiation of Cerion: a remarkably diverse group of

West Indian land snails. *Malacologia* 17:223–239. [www.biodiversitylibrary.org/page/13092786](http://www.biodiversitylibrary.org/page/13092786).

Woodruff DS, Gould SJ. 1980. Geographic differentiation and speciation in Cerion-a preliminary

discussion of patterns and processes. *Biological Journal of the Linnean Society* 14(3–4):389–416

DOI 10.1111/j.1095-8312.1980.tb00115.x.
