# Supplementary material for: Adapting mark-recapture methods to estimating accepted species-level diversity: a case study with terrestrial Gastropoda
Source: PeerJ. 2022 Jun 21;10:e13139. doi: 10.7717/peerj.13139 (PMC9231345; doi:10.7717/peerj.13139)
Supplement: Supplemental Information 5 — Geographic distribution of names sampled at ANSP, showing percent accepted names missing from MolluscaBase by region. Raw data are in Tables S2 and S3. [file peerj-10-13139-s005.docx]

| **August 2020** | Africa | Asia | Austra-lia/NZ | Carib- bean | Europe | North America | Oceania | South America | Other | Total |
| --- | --- | --- | --- | --- | --- | --- | --- | --- | --- | --- |
| **Total Names** | 130 | 308 | 44 | 140 | 129 | 147 | 115 | 82 | 5 | 1100 |
| **Excluded** | 5 | 8 | 0 | 11 | 6 | 25 | 3 | 1 | 2 | 61 |
| Marine | 0 | 0 | 0 | 0 | 0 | 0 | 0 | 0 | 1 | 1 |
| Fossil only | 1 | 1 | 0 | 1 | 3 | 0 | 0 | 0 | 0 | 6 |
| Not available | 3 | 4 | 0 | 2 | 2 | 1 | 1 | 1 | 1 | 15 |
| Cerionidae | 0 | 0 | 0 | 6 | 0 | 20 | 0 | 0 | 0 | 26 |
| Truncatellidae | 0 | 0 | 0 | 2 | 0 | 0 | 1 | 0 | 0 | 3 |
| Duplicate | 1 | 3 | 0 | 0 | 1 | 4 | 1 | 0 | 0 | 10 |
| Total included [n_2_] | 125 | 300 | 44 | 129 | 123 | 122 | 112 | 81 | 3 | 1039 |
|  |  |  |  |  |  |  |  |  |  |  |
| Epithet and accepted name present | 94 | 270 | 44 | 50 | 103 | 118 | 64 | 72 | 3 | 818 |
| Epithet present, accepted name uncertain/unknown | 1 | 1 | 0 | 0 | 1 | 0 | 0 | 0 | 0 | 3 |
| Epithet present, accepted name missing | 0 | 1 | 0 | 3 | 1 | 0 | 0 | 0 | 0 | 5 |
| Epithet missing; accepted name present | 3 | 3 | 0 | 1 | 13 | 2 | 0 | 1 | 0 | 23 |
| Epithet missing, status uncertain/unknown | 5 | 5 | 0 | 1 | 3 | 0 | 2 | 0 | 0 | 16 |
| Epithet missing; accepted name missing | 22 | 20 | 0 | 74 | 2 | 2 | 46 | 8 | 0 | 174 |
| **Percent accepted names missing** | **17.6** | **6.7** | **0.0** | **57.4** | **1.6** | **1.6** | **41.1** | **9.9** | **0.0** | **16.7** |
| **July 2021** | | | | | | | | | | |
| **Total Names** | 132 | 304 | 31 | 145 | 131 | 140 | 118 | 95 | 4 | 1100 |
| **Excluded** | 8 | 5 | 1 | 14 | 5 | 28 | 3 | 0 | 0 | 64 |
| Marine | 0 | 0 | 0 | 0 | 0 | 0 | 0 | 0 | 0 | 0 |
| Fossil only | 1 | 0 | 0 | 0 | 1 | 0 | 0 | 0 | 0 | 2 |
| Not available | 1 | 3 | 0 | 2 | 2 | 1 | 2 | 0 | 0 | 11 |
| Cerionidae | 0 | 0 | 0 | 8 | 0 | 26 | 0 | 0 | 0 | 34 |
| Truncatellidae | 1 | 1 | 0 | 4 | 0 | 1 | 1 | 0 | 0 | 8 |
| Duplicate | 5 | 1 | 1 | 0 | 2 | 0 | 0 | 0 | 0 | 9 |
| Total included [n_2_] | 124 | 299 | 30 | 131 | 126 | 112 | 115 | 95 | 4 | 1036 |
|  |  |  |  |  |  |  |  |  |  |  |
| Epithet and accepted name present | 110 | 287 | 30 | 103 | 117 | 104 | 100 | 88 | 4 | 943 |
| Epithet present, accepted name uncertain/unknown | 0 | 0 | 0 | 0 | 0 | 0 | 0 | 0 | 0 | 0 |
| Epithet present; accepted name missing | 2 | 2 | 0 | 0 | 1 | 2 | 3 | 0 | 0 | 10 |
| Epithet missing; accepted name present | 3 | 3 | 0 | 1 | 3 | 6 | 1 | 2 | 0 | 19 |
| Epithet missing, status uncertain/unknown | 1 | 2 | 0 | 0 | 5 | 0 | 1 | 0 | 0 | 9 |
| Epithet missing; accepted name missing | 8 | 5 | 0 | 27 | 0 | 0 | 10 | 5 | 0 | 55 |
| **Percent accepted names missing** | **6.5** | **1.7** | **0.0** | **20.6** | **0.0** | **0.0** | **8.7** | **5.3** | **0.0** | **5.3** |
