# Supplementary material for: Adapting mark-recapture methods to estimating accepted species-level diversity: a case study with terrestrial Gastropoda
Source: PeerJ. 2022 Jun 21;10:e13139. doi: 10.7717/peerj.13139 (PMC9231345; doi:10.7717/peerj.13139)
Supplement: Supplemental Information 6 — Taxonomic distribution of names sampled at ANSP, showing percent accepted names missing from MolluscaBase by clade (or grade, i.e., operculates). Raw data are in Tables S2 and S3. [file peerj-10-13139-s006.docx]

**Table S4.** Taxonomic distribution of names sampled at ANSP, showing percent accepted names missing from MolluscaBase by clade (or grade, i.e., operculates). Raw data is in Tables S1 and S2.

| **August 2020** | **Eupul-monata** | **Neriti-morpha** | **Cyclophor-oidea** | **Littorini-morpha** | **All Oper** **cul** **ates** | **Total** |
| --- | --- | --- | --- | --- | --- | --- |
| **Total Names** | 917 | 36 | 106 | 41 | 183 | 1100 |
| **Excluded** | 52 | 0 | 6 | 3 | 9 | 61 |
| Marine | 1 | 0 | 0 | 0 | 0 | 1 |
| Fossil only | 5 | 0 | 1 | 0 | 1 | 6 |
| Not available | 12 | 0 | 3 | 0 | 3 | 15 |
| Cerionidae | 26 | 0 | 0 | 0 | 0 | 26 |
| Truncatellidae | 0 | 0 | 0 | 3 | 3 | 3 |
| Duplicate | 8 | 0 | 2 | 0 | 2 | 10 |
| Total included [n_2_] | 865 | 36 | 100 | 38 | 174 | 1039 |
|  |  |  |  |  |  |  |
| Epithet and accepted name present | 679 | 26 | 95 | 18 | 139 | 818 |
| Epithet present, accepted name uncertain/unknown | 3 | 0 | 0 | 0 | 0 | 3 |
| Epithet present, accepted name missing | 4 | 0 | 1 | 0 | 1 | 5 |
| Epithet missing; accepted name present | 23 | 0 | 0 | 0 | 0 | 23 |
| Epithet missing, status uncertain/unknown | 14 | 2 | 0 | 0 | 2 | 16 |
| Epithet missing; accepted name missing | 142 | 8 | 4 | 20 | 32 | 174 |
| **Percent missing accepted names** | **16.4** | **22.2** | **4.0** | **52.6** | **18.4** | **16.7** |
| **July 2021** | | | | | | |
| **Total Names** | 876 | 50 | 105 | 69 | 224 | 1100 |
| **Excluded** | 51 | 3 | 1 | 9 | 13 | 64 |
| Marine | 0 | 0 | 0 | 0 | 0 | 0 |
| Fossil only | 2 | 0 | 0 | 0 | 0 | 2 |
| Not available | 6 | 3 | 1 | 1 | 5 | 11 |
| Cerionidae | 34 | 0 | 0 | 0 | 0 | 34 |
| Truncatellidae | 0 | 0 | 0 | 8 | 8 | 8 |
| Duplicate | 9 | 0 | 0 | 0 | 0 | 9 |
| Total included [n_2_] | 825 | 47 | 104 | 60 | 211 | 1036 |
|  |  |  |  |  |  |  |
| Epithet and accepted name present | 750 | 40 | 104 | 49 | 193 | 943 |
| Epithet present, accepted name uncertain/unknown | 0 | 0 | 0 | 0 | 0 | 0 |
| Epithet present, accepted name missing | 8 | 2 | 0 | 0 | 2 | 10 |
| Epithet missing; accepted name present | 18 | 2 | 0 | 0 | 2 | 20 |
| Epithet missing, status uncertain/unknown | 6 | 1 | 0 | 1 | 2 | 8 |
| Epithet missing; accepted name missing | 43 | 2 | 0 | 10 | 12 | 55 |
| **Percent missing accepted names** | **5.2** | **4.3** | **0.0** | **16.7** | **5.7** | **5.3** |
